# Insights of mammalian hibernator-derived cholangiocyte organoids in improving liver cold preservation

**DOI:** 10.1093/procel/pwaf052

**Published:** 2025-07-01

**Authors:** Chuman Wu, Changliang Wang, Meifeng Gu, Weiya He, Wenjun Deng, Wenjie Huang, Jiayu Liao, Changhui Li, Weilue Chen, Ruiping Chen, Ji Dong, Meiling Liu

**Affiliations:** GMU–GIBH Joint School of Life Sciences, Guangzhou Medical University, Guangzhou 511436, China; Guangzhou National Laboratory, Guangzhou 510005, China; Guangzhou National Laboratory, Guangzhou 510005, China; Guangzhou National Laboratory, Guangzhou 510005, China; Innovation Center for Evolutionary Synthetic Biology, School of Life Sciences, Sun Yat-sen University, Guangzhou 510275, China; Faculty of Health Sciences and UM–Bioland Joint Laboratory, University of Macau, Macao 999078, China; Innovation Center for Evolutionary Synthetic Biology, School of Life Sciences, Sun Yat-sen University, Guangzhou 510275, China; Guangzhou National Laboratory, Guangzhou 510005, China; GMU–GIBH Joint School of Life Sciences, Guangzhou Medical University, Guangzhou 511436, China; Guangzhou National Laboratory, Guangzhou 510005, China; Guangzhou National Laboratory, Guangzhou 510005, China; Guangzhou National Laboratory, Guangzhou 510005, China; Department of Thoracic Surgery, The First Affiliated Hospital of Sun Yat-sen University, Guangzhou 510080, China; GMU–GIBH Joint School of Life Sciences, Guangzhou Medical University, Guangzhou 511436, China; Guangzhou National Laboratory, Guangzhou 510005, China; Innovation Center for Evolutionary Synthetic Biology, School of Life Sciences, Sun Yat-sen University, Guangzhou 510275, China

## Dear Editor,

As a formidable health-care burden, liver disease affects approximately 844 million people worldwide, and around 2 million patients with liver disease die per year (Marcellin and Kutala, 2018). For patients suffering from end-stage liver disease, liver transplantation is the most ideal and final treatment (Ferreira-Gonzalez et al., 2022). Unfortunately, up to 35% of the recipients after liver transplantation are subjected to biliary complications (Ferreira-Gonzalez et al., 2022), such as anastomotic (AS), non-anastomotic biliary strictures (NAS), and bile leakage, which represent the major causes of morbidity and graft failure after liver transplantation (Brunner et al., 2013). One of the major reasons is that, compared with other liver parenchymal cells, cholangiocytes are more vulnerable to the effects of static cold storage (SCS) (Ferreira-Gonzalez et al., 2022), the standard clinical method of organ preservation. SCS results in the deterioration of the biliary tract, such as the loss of biliary epithelia, mural necrosis, and the damage of peribiliary vascular plexus (Brunner et al., 2013). Although more advanced preservation techniques are being developed, such as normo- or hypothermic *ex situ* perfusion technologies, the improvement of biliary complications is limited (Nasralla et al., 2018).

In nature, many mammals can hibernate to survive harsh conditions with food shortage and low ambient temperature (Mohr et al., 2020). During hibernation, small mammalian hibernators, such as Syrian hamsters, ground squirrels, chipmunks, bats, and so on, can reduce their body temperatures to below 10°C from ~37°C normothermia and repeat many torpor-arousal cycles (Carey et al., 2003). Thus, their organs are subjected to repetitive cooling-rewarming stresses, yet can be shielded from such detrimental insults (Dugbartey et al., 2018). Recent studies have shown that hibernator-derived cells are more tolerant to cold stress than cells derived from nonhibernators (e.g., humans and mice) (Anegawa et al., 2021; Ou et al., 2018; Sone et al., 2024). However, these studies mainly depend on primary cells or immortalized cell lines, which cannot fully reflect the cold adaptation mechanism at the level of *in vivo* tissues.

Liver-derived cholangiocyte organoids serve as an ideal model for biliary epithelial cells, as they possess the remarkable capacity of self-organization and can maintain the majority of biliary characteristics during *in vitro* culture (Huch et al., 2015). Several studies have proved the superiority of cholangiocyte organoids in modeling biliary cold storage, ischemia and reperfusion injury, and recapitulating cholangiopathy-associated programmed cell death (Ferreira-Gonzalez et al., 2022). In this study, we attempted to build intrahepatic cholangiocyte organoids (ICOs) from the mammalian hibernator, Syrian hamster (*Mesocricetus auratus*), and performed a comparison between Syrian hamster ICOs (shICOs) and mouse ICOs (mICOs) to seek for molecular clues of biliary cold adaptation.

**Figure 1.**
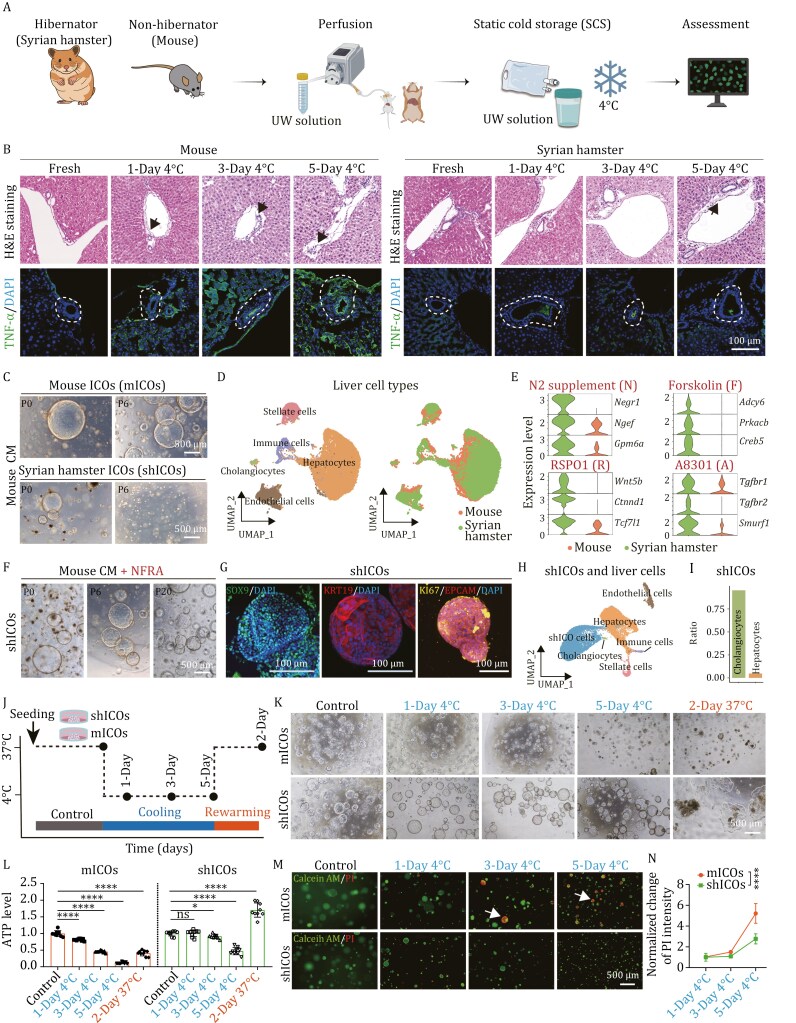
The establishment of the culture system of Syrian hamster intrahepatic cholangiocyte organoids (shICOs). (A) Schematic diagram depicting the procedures of perfusion with the University of Wisconsin (UW) solution and static cold storage (SCS) of Syrian hamster and mouse livers. (B) H&E staining and IF staining for TNF-α in mouse and Syrian hamster liver tissues after 1, 3, and 5 days of 4°C SCS, with freshly resected livers serving as the control group. Black arrow indicates compromised biliary architectures. White dash line indicates bile ducts. (C) Representative phase-contrast images of shICOs cultured in mouse culture medium (CM), which failed to support shICOs, with mICOs serving as controls. (D) Uniform manifold approximation and projection (UMAP) visualization of liver cell clusters of mice (*n* = 2) and Syrian hamsters (*n* = 2) based on 26,384 single nuclei. (E) Violin plots showing the expression differences of representative genes related to neuronal development, cAMP, Wnt, and TGF-β signaling pathways in Syrian hamster and mouse cholangiocytes. (F) Representative phase-contrast microscopy images of shICOs at early, intermediate and late passages (e.g., P0, P6, and P20), demonstrating consistent morphology throughout long-term culture. (G) Representative IF microscopy images of shICOs at P10 demonstrate the expression of biliary markers SOX9, KRT19, and EPCAM, along with the proliferation marker KI67, indicating robust proliferation. (H) UMAP visualization of the integration of shICO cells and Syrian hamster liver cells. (I) The cell type ratio of shICOs. (J) Diagram illustrating the cooling-rewarming experiments for mICOs and shICOs. (K) Representative images of cell morphology showing that shICOs maintained more intact cellular structures under cold stress and exhibited robust proliferation upon rewarming, whereas mICOs presented severe cell damage and death during the cooling-warming process. (L) Cell viability in shICOs and mICOs assessed by the ATP assays during the cooling-rewarming process. Data are presented as mean ± standard deviation. ****P < 0.0001; ***P < 0.001; *P < 0.05; ns *P* > 0.05, not significant (*t*-test). (M) Calcein AM/propidium iodide (PI) staining of live (green) and dead (red) cells in shICOs and mICOs cultured at 37°C (control) and at 4°C for 1, 3, and 5 days. White arrow indicates dead cells. (N) Quantitative analysis of PI levels in shICOs and mICOs under cold stress. Data are presented as mean ± standard deviation. ****P < 0.0001 (two-way ANOVA).

We first determined whether Syrian hamster cholangiocytes could cope with cold stress better than their non-hibernator (i.e., mouse) counterparts could. Using the University of Wisconsin (UW) solution, the standard organ cold preservation solution in clinical practice, we perfused livers of Syrian hamsters and mice and preserved them under SCS at 4°C for different times (i.e., 1, 3 and 5 days) (Fig. 1A). The hematoxylin and eosin (H&E) staining indicated that mouse livers after SCS exhibited severe cold-induced injuries, with focal necrotic areas of hepatocytes, pronounced cellular edema, substantial disruption of the lobular architecture, and particularly the peeling of periportal cells (Figs. 1B and S1A). Moreover, the immunohistochemical (IHC) staining and immunofluorescence (IF) staining for the cholangiocyte marker Keratin19 (KRT19) revealed that the morphology of mouse bile ducts was progressively altered with the extension of SCS (Fig. S1B and S1C). Mouse bile ducts suffered from impaired duct continuity, cell death, and detachment of cholangiocytes into the lumen, akin to those observed in human NAS. The IF staining for inflammatory markers TNF-α and CD68 further demonstrated that the mouse liver (especially cholangiocytes and hepatocytes) presented a pronounced inflammatory response since the first day of SCS (Figs. 1B and S1C). In contrast, the Syrian hamster liver preserved a well-maintained tissue structure and morphology, and no severe bile duct damage and evident inflammatory response were detected even after 5 days of SCS. These results imply that Syrian hamster cholangiocytes exhibit a better cold resistance ability than their mouse counterparts do during SCS, which also suggests Syrian hamster as an ideal model for studying organ cold adaptation.

Next, we attempted to construct shICOs to characterize the cold adaptation mechanisms of Syrian hamster bile ducts. However, the direct use of mouse culture medium failed to support shICOs (Fig. 1C). To identify the molecular differences between Syrian hamster and mouse cholangiocytes, we used single-nucleus RNA sequencing (snRNA-seq) to profile liver transcriptomes of these two species, each with two replicates. In total, 26,384 single nuclei were obtained, and five major cell groups were identified, namely, hepatocytes, cholangiocytes, stellate cells, endothelial cells, and immune cells (Figs. 1D and S2A–D; Table S1). We then performed differential expression analysis and detected a clear difference between Syrian hamster and mouse cholangiocytes (Fig. S2E; Table S2). Gene ontology analysis demonstrated that the upregulated genes of Syrian hamster cholangiocytes were predominantly enriched in terms associated with neuronal development, cAMP, Wnt, and TGF-β signaling pathways (Figs. 1E and S2E). However, the high expression level of *Smurf1* indicated the inhibition of TGF-β signaling in Syrian hamster cholangiocytes, as *Smurf1* encodes a ubiquitin ligase that can ubiquitinate and target TGFBR1 for degradation (Yan et al., 2011).

Given the above results, we successfully established the culture system of shICOs by adding a composition combination termed as NFRA (N: N2 supplement, for neuronal development; F: forskolin, a cAMP activator; R: R-spondin-1 conditioned medium, for Wnt activation; A: A8301, a TGF-β inhibitor) to the mouse culture medium, and the removal of any one of the four factors would lead to the failure of shICO culture (Figs. 1F and S2F). Notably, shICOs manifested as spherical structures composed of a single layer of cubical epithelium, which could be maintained for a long-term expansion, such as over 20 passages.

Furthermore, the IHC and IF staining for cholangiocyte markers, such as SOX9, KRT19 and EPCAM, suggested their high expression levels in shICOs (Figs. 1G  S3A and S3B). Meanwhile, the IF staining for MKI67 also supported the proliferative feature of shICOs. In addition, we conducted snRNA-seq to dissect the cell composition of shICOs and obtained 11,928 single nuclei (Fig. S3C). The integration result of shICO cells and *in vivo* liver cells revealed that more than 90% of shICO cells were grouped with *in vivo* cholangiocytes, while other shICO cells were grouped with *in vivo* hepatocytes (Fig. 1H and 1I). Notably, shICO cells also exhibited consistent expression patterns of marker genes with *in vivo* hepatocytes (e.g., *Hnf4a* and *Tfr2*) and cholangiocytes (e.g., *Epcam* and *Pkhd1*) (Fig. S3D and S3E; Table S3). Taken together, these results demonstrate the success of the culture system of shICOs.

Subsequently, we investigated whether shICOs could recapitulate the cold resistance ability of Syrian hamster cholangiocytes. We utilized shICOs to simulate the SCS and reperfusion processes during liver transplantation by cooling (4°C) and rewarming (37°C) experiments, and mICOs were used as controls (Fig. 1J). shICOs and mICOs were initially cultured at 37°C under normal conditions, then were transferred to a cooling condition at 4°C, and finally were rewarmed back to 37°C under normal conditions. Importantly, remarkable differences between shICOs and mICOs were observed (Fig. 1K). After 5 days of cooling, the vast majority of mICO spheroids underwent shrinkage and turned black, and almost no mICOs survived after 2 days of rewarming. In contrast, shICOs were able to preserve their spheroid morphology under cold stress and promptly resume proliferation with cells adhering to the culture vessel wall upon rewarming.

Furthermore, we performed ATP assay to evaluate the cell viability of shICOs and mICOs (Fig. 1L). Upon cooling, ATP levels gradually decreased in mICOs, and only less than 10% was left in mICOs after 5 days of cooling. On the other hand, shICOs only exhibited a sharp decrease of ATP levels after 5 days of cooling, but still maintained a 50% level, and quickly recovered upon rewarming. In addition, propidium iodide (PI) staining of dead cells indicated that 3 days of cooling at 4°C induced widespread cell death in mICOs but not in shICOs (Figs. 1M, 1N and S4A). Therefore, these results indicate that shICOs present superior resistance ability to cooling-rewarming stress compared with mICOs.

**Figure 2.**
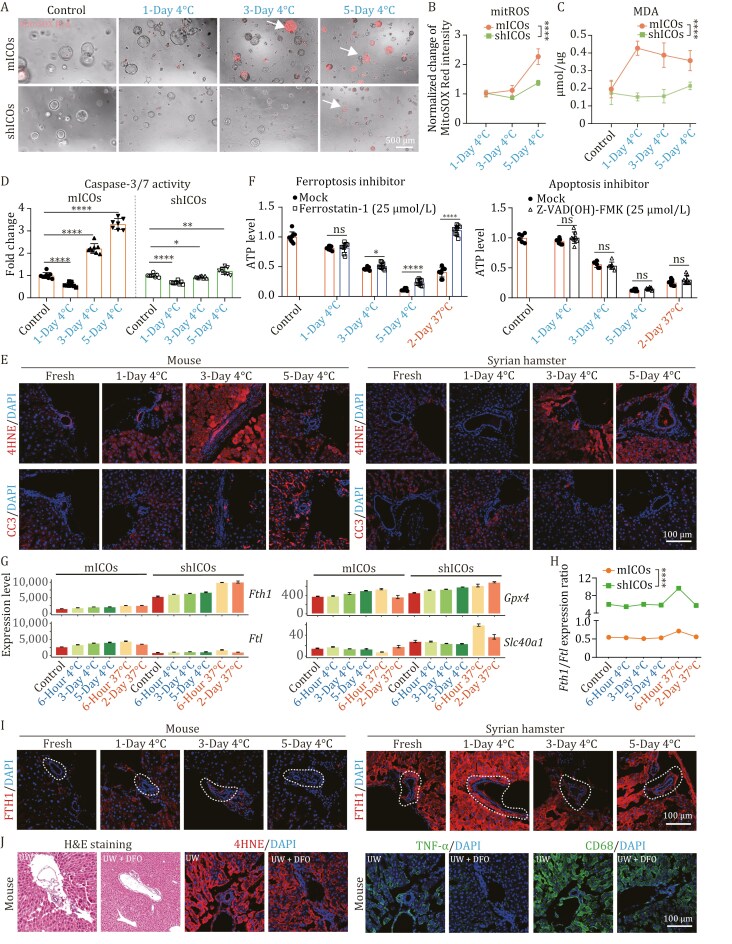
The superior anti-ferroptosis ability of shICOs under cold stress. (A) Phase-contrast imaging of shICOs and mICOs, merged with MitoSOX Red staining, which evaluates the levels of cold-induced mitochondrial ROS (mitROS). White arrow indicates cells with high levels of mitochondrial ROS. (B) Quantitative analysis of mitROS levels in shICOs and mICOs under cold stress. Data are presented as mean ± standard deviation. ****P < 0.0001 (two-way ANOVA). (C) Lipid peroxidation in shICOs and mICOs evaluated by the relative MDA levels. Data are presented as mean ± standard deviation. ****P < 0.0001 (two-way ANOVA). (D) Caspase-3/7 activity in shICOs and mICOs at 37°C and after exposure to cold stress at 4°C for 1, 3, and 5 days. ****P < 0.0001; ***P < 0.001; *P < 0.05 (*t*-test). (E) IF staining for 4-Hydroxynonenal (4HNE, an oxidative/nitrosative stress biomarker) and Cleaved Caspase-3 (CC3, an apoptosis biomarker) in mouse and Syrian hamster liver tissues following 4°C SCS at 1, 3, and 5 days, with freshly resected livers serving as the control group. (F) Cell viability in mICOs assessed by the ATP assays during the cooling-rewarming process, with treatments of Ferrostatin-1 (25 μmol/L) to inhibit ferroptosis or the pan-caspase inhibitor Z-VAD(OH)-FMK (25 μmol/L) to inhibit apoptosis. Data are presented as mean ± standard deviation. ****P < 0.0001; ***P < 0.001; *P < 0.05; ns *P* > 0.05, not significant (*t*-test). (G) Barplots showing the expression levels of *Fth1*, *Ftl*, *Gpx4,* and *Sla40a1* in shICOs and mICOs at 6 time points during the cooling-rewarming process. (H) The comparison of *Fth1*/*Ftl* expression ratio between mICOs and shICOs during the cooling-rewarming process. ****P < 0.0001 (two-way ANOVA). (I) IF staining for FTH1 in mouse and Syrian hamster liver tissues following 4°C SCS at 1, 3, and 5 days, with freshly resected livers serving as the control group. White dash line indicates bile ducts. (J) H&E staining and IF staining for 4HNE, TNF-α, and CD68 showing improved cold preservation of mouse bile ducts in UW solution with deferoxamine (DFO, 100 μmol/L) after 5 days of 4°C SCS, with the standard UW solution as the control group.

Recent investigations have indicated that the cell death triggered by cold exposure manifests the hallmarks of ferroptosis, which is typified by substantial lipid peroxidation facilitated through iron-ion-mediated generation of reactive oxygen species (ROS) (Sone and Yamaguchi, 2024). Thus, we first assessed the levels of ROS by the live cell fluorescent probe MitoSOX Red that targets mitochondrial superoxide. A significant increase in ROS production was observed in mICOs but not in shICOs after 3 days of cooling at 4°C (Fig. 2A and 2B). We further measured the levels of malondialdehyde (MDA) to assess lipid peroxidation (Fig. 2C). Notably, within the initial day of cold exposure, intense lipid peroxidation was detected in mICOs, with a more than 2-fold elevation in the MDA concentration. Surprisingly, even after 5 days of cooling, the MDA concentration in shICOs was still maintained at a low level. In addition, we also measured the levels of apoptosis with caspase-3/7 activity in both shICOs and mICOs under cold stress (Fig. 2D). Throughout the cooling process, shICOs exhibited no evident apoptosis, whereas mICOs showed significant apoptosis starting from the third day of cold exposure.

To determine whether the results of cold stress-induced cell death obtained from *in vitro* organoids could truly reflect the results at the *in vivo* tissue level, we then evaluated the levels of lipid peroxidation and apoptosis in liver tissues of hamsters and mice following 1-, 3-, and 5-day SCS (Fig. 2E). According to the IF staining results for lipid peroxidation marker 4-Hydroxynonenal (4HNE) and apoptosis marker Cleaved Caspase-3 (CC3), mouse livers experienced lipid peroxidation and apoptosis within 1-day and 3-day SCS, respectively, indicating that lipid peroxidation occurs earlier than apoptosis in mouse livers. In contrast, for Syrian hamster livers, lipid peroxidation occurred at a later stage of SCS, and no apoptotic signs were detected even after 5 days of SCS. Furthermore, we examined the impacts of the ferroptosis inhibitor Ferrostatin-1 (Fer-1) and the apoptosis inhibitor Z-VAD(OH)-FMK on the cell viability of mICOs under cooling-rewarming stress (Figs. 2F and S4B). Unlike the weak protective effect of Z-VAD(OH)-FMK, Fer-1 substantially augmented the cell viability of mICOs under cold stress and rapidly assisted mICOs in recovering their vitality after rewarming. To sum up, although mICOs encountered both ferroptosis and apoptosis under cold stress, ferroptosis occurred much earlier and had a greater impact on the survival rate of mICOs. Compared with mICOs, shICOs were able to maintain low levels of ROS and lipid peroxidation, suggesting a better anti-ferroptosis ability.

To explore the molecular mechanisms underlying the disparity in anti-ferroptosis ability between shICOs and mICOs, their transcriptomes were profiled at 6 time points, namely, 37°C normal condition, 6-hour, 3-day, and 5-day 4°C cooling condition, and 6-hour and 2-day 37°C rewarming condition (Fig. S5A). We investigated the expression patterns of the marker genes that are related to the canonical ferroptosis pathway (Fig. S5B). Importantly, aside from the moderate upregulation of the renowned anti-lipid peroxidation gene, *Gpx4*), shICOs also exhibited a much higher expression level of *Fth1* that encodes the heavy subunit of ferritin (Fig. 2G). Ferritin stores iron in a form that is not only soluble and non-toxic but also easily retrievable, which is crucial in maintaining iron homeostasis (Zhang et al., 2021). Moreover, ferritin consists of 24 subunits of the heavy (FTH) and light (FTL) chains, and the variation in the subunit composition may affect its function. Interestingly, the expression level of *Ftl* that encodes the light subunit of ferritin was much lower in shICOs than in mICOs, resulting in a remarkably distinct FTH/FTL ratio of ferritin between shICOs and mICOs (Fig. 2H). As ferritin with more heavy chains has a stronger antioxidant activity (Zhang et al., 2021), the higher FTH/FTL ratio of ferritin in shICOs might suggest their superior antioxidant ability during the cooling-rewarming process.

Furthermore, we also conducted IF staining for FTH1 in mouse and Syrian hamster liver tissues during SCS (Fig. 2I). Notably, FTH1 exhibited much higher expression levels in Syrian hamster livers than mouse livers throughout the SCS process, which was consistent with the organoid results. In addition, shICOs more highly expressed *Slc40a1* (Fig. 2G), which encodes an ion transport protein that can transport Fe^2+^ from the intracellular to the extracellular space and is also crucial to the maintenance of iron homeostasis (Zhang et al., 2018). Based on the higher expression of *Fth1* and *Slc40a1*, we inferred a better maintenance of iron homeostasis in shICOs. Thus, we improved the UW solution by adding the iron chelator, deferoxamine (DFO), which might help to chelate the cellular free iron and reduce the generation of toxic ROS. Compared with the standard UW solution, UW solution with DFO indeed improved the cold preservation of mouse bile ducts, with better duct continuity, less detachment of cholangiocytes into the lumen, and the alleviation of ferroptosis and inflammation (Figs. 2J and S5C). Similarly, DFO treatment also improved the cold preservation of rat bile ducts (Fig. S6).

In conclusion, using the mammalian hibernator, Syrian hamster, as an animal model, we demonstrated the superior cold resistance ability in their cholangiocytes and recapitulated such ability *in vitro* by building the culture system of shICOs. Recently, several studies have utilized hibernator-derived cells to study cellular cold tolerance (Anegawa et al., 2021; Ou et al., 2018; Sone et al., 2024); however, these cells are much inferior in the self-organization capacity and the maintenance of *in vivo* characteristics compared with organoids. We further demonstrated the stronger anti-ferroptosis ability of shICOs than mICOs under cold stress. Although cold exposure-induced cell death exhibits the characteristics of ferroptosis (Sone and Yamaguchi, 2024), the results obtained by different systems are not exactly the same (Anegawa et al., 2021; Sone et al., 2024). The cross-species comparison between shICOs and mICOs revealed that, in addition to *Gpx4*, *Fth1* and *Slc40a1* were also upregulated in shICOs during the cooling-rewarming process, implying the importance of iron homeostasis in biliary cold adaptation. Collectively, our findings provide unique insights into improving biliary cold preservation, and our shICO model will be a useful tool for the future study of mammalian hibernation.

## Supplementary data

Supplementary data is available at *Protein & Cell* online https://doi.org/10.1093/procel/pwaf052.

pwaf052_suppl_Supplementary_Figures_S1-S6

pwaf052_suppl_Supplementary_Table_S1

pwaf052_suppl_Supplementary_Table_S2

pwaf052_suppl_Supplementary_Table_S3

## Footnotes

We would like to thank the Advanced Drug Discovery Platform of Guangzhou National Laboratory for our cell scanning work and we would be grateful to Jinfeng Luo for her help of analyzing images on the Operetta CLS system. This work was supported by the Strategic Priority Research Program of the Chinese Academy of Sciences (Grant No. XDB1030000), the National Natural Science Foundation of China (32422027) and the Research Foundation for Advanced Talents from Guangzhou National Laboratory (YW-JCYJ0604).

J.D. and M.L.L. conceived and designed the study; C.M.W., M.F.G., W.Y.H., W.J.D., W.J.H., J.Y.L., C.H.L., W.L.C. and R.P.C. performed the experiments. C.L.W. conducted the bioinformatics analyses. J.D. and C.M.W. wrote the manuscript with help from all the authors. All authors reviewed the manuscript.

The authors declare no competing interests.

This study was approved by the Administrative Panel on Laboratory Animal Care at the Guangzhou National Laboratory (Guangzhou, China). The authors confirm their willingness to participate and declare their agreement to publish.

All data generated in this study are deposited in the Genome Sequence Archive (GSA) database (accession number: CRA021820). Other resources are available on request.
