# Supplementary material for: Insights of mammalian hibernator-derived cholangiocyte organoids in improving liver cold preservation
Source: Protein Cell. 2025 Jul 1;17(8):790–6. doi: 10.1093/procel/pwaf052 (PMC13429465; doi:10.1093/procel/pwaf052)
Supplement: pwaf052_suppl_Supplementary_Figures_S1-S6 [file pwaf052_suppl_supplementary_figures_s1-s6.pdf]

# Supplemental Materials

## Insights of mammalian hibernator-derived cholangiocyte organoids in improving liver cold preservation

**Authors:** Chuman Wu,[1,2,†] Changliang Wang,[2,†] Meifeng Gu,[2,3,†] Weiya He,[4,†] Wenjun Deng,[3] Wenjie Huang,[2] Jiayu Liao,[1,2] Changhui Li,[2] Weilue Chen,[2] Ruiping Chen,[5] Ji Dong,[1,2,*] Meiling Liu[3,*]

**Author Affiliations:**

[1]GMU-GIBH Joint School of Life Sciences, Guangzhou Medical University, Guangzhou 511436, China

[2]Guangzhou National Laboratory, Guangzhou 510005, China

[3]Innovation Center for Evolutionary Synthetic Biology, School of Life Sciences, Sun Yat-sen University, Guangzhou 510275, China

[4]Faculty of Health Sciences and UM-Bioland Joint Laboratory, University of Macau, Macau 999078, China

[5]Department of Thoracic Surgery, The First Affiliated Hospital of Sun Yat-sen University, Guangzhou, Guangdong, 510080, China.

[†]These authors contributed equally to this work: Chuman Wu, Changliang Wang, Meifeng Gu, Weiya He.

[*]Correspondence: dong_ji@gzlab.ac.cn (J.D.); liumling33@mail.sysu.edu.cn (M.L.).

**This file includes:**

Materials and Methods

Supplemental Figure Legends

Supplemental Table Legends

## Materials and Methods

### Animals

In this study, male Syrian hamsters aged 12-16 weeks, male C57BL/6J mice aged 8-12 weeks and male Sprague-Dawley rats aged 10 weeks were used. The Syrian hamsters were procured from Charles River Laboratories (China) and were individually housed in plastic cages at the animal facility of Guangzhou National Laboratory. They were kept in a meticulously controlled environment, maintained at an ambient temperature of 22°C with a precisely regulated 12:12-hour light-dark cycle. The mice and rats, which were purchased from Casgene Biotech CO., Ltd (China) and Guangzhou Qingle Life Sciences Co., Ltd (China), respectively, were reared under conditions that were identical to those of the Syrian hamsters. All animals were provided with unrestricted (ad libitum) access to water and a standard rodent diet. This study was approved by the Administrative Panel on Laboratory Animal Care at the Guangzhou National Laboratory (Guangzhou, China).

### *Ex vivo* liver perfusion and cold storage

Before surgery, Syrian hamsters, mice and rats were given deep subcutaneous heparin (0.5 units/g) injections in the lower abdominal fat layer. Subsequently, animals were placed in a supine position after being anesthetized by injection with urethane. To prevent hypothermia, they were placed on an electric warming plate set at 40°C. Next, via an abdominal incision, the intestines were carefully moved aside to expose the liver portal and the inferior vena cava. Liver perfusion was carried out retroactively, from the inferior vena cava to the portal vein. A 26G cannula was used for mice and rats, while a 22G cannula was used for hamsters, inserted into the vena cava. The mouse livers were then perfused with 15-20 ml of University of Wisconsin (UW) solution over a span of 5 minutes, and the hamster and rat livers with 30-50 ml of UW solution over 10 minutes. The perfusion process continues until the color of the organs changes from dark brown to pale, indicating the completion of *in situ* perfusion (Giacalone et al., 2022). Following this, the mouse livers were excised and stored in 10 ml of UW

preservation solution, and the hamster and rat livers were stored in 25 ml of UW preservation solution, within sterile plastic tubes at 4°C for 1, 3, or 5 days. In addition, to test the protective effect of deferoxamine (DFO) on the cold preservation of mouse and rat bile ducts, UW solution was supplemented with DFO (HY-B0988, MedChemExpress) at 100 μM or 1 mM concentrations.

**Isolation and culture of mouse and Syrian hamster intrahepatic cholangiocyte organoids (ICOs)**

As previously described, mice and Syrian hamsters were processed following a well-established protocol (Broutier et al., 2016). In compliance with approved institutional guidelines, we sacrificed the animals and harvested their livers, immediately transferring them to a dish with cold DMEM/F12 medium. The biliary ducts were then prepared from the harvested livers, adhering to methods from prior studies with slight adaptations. Post mechanical dissection, the liver tissues were enzymatically digested in DMEM/F12 medium (Gibco) supplemented with 0.3 mg/ml type IV collagenase (Gibco) and 0.3 mg/ml dispase (Gibco) at 37°C for 3 to 5 hours, until biliary duct fragments were visibly separated. The isolated ducts were subsequently mixed with Matrigel (BD Biosciences) and seeded. Once the Matrigel had solidified, culture medium was added to initiate the culture process.

Initially, both mouse ICOs (mICOs) and Syrian hamster ICOs (shICOs) were cultured in mouse HepatiCult™ Organoid Growth Medium (OGM) (STEMCELL Technologies, 06030) for testing. Later, we optimized the medium, the culture medium for shICOs was switched to OGM supplemented with NFRA, which consists of 100x N2 supplement, 5μM Forskolin (an adenylyl cyclase activator), 5% RSPO condition medium, and 5 μM A8301 (an FGFR inhibitor).

**Cell viability assays, lipid peroxidation and cell apoptosis detection**

ICOs viability was assessed using the CellCounting-Lite 3D Luminescent Cell Viability

Assay (Vazyme, DD1102). ICOs cultured in 96-well plates were treated with 100 μl of the CellCounting-Lite 3D reagent per well. The plates were then vigorously agitated for 10 minutes on a microplate shaker to ensure complete cell lysis. Following a 30-minute incubation at room temperature in the dark, luminescence was measured (Pendergraft et al., 2017).

The degree of lipid peroxidation is determined through the quantification of Malondialdehyde (MDA) levels were quantified using the MDA Fluorometric Assay Kit (Elabscience, E-BC-F007) in accordance with the manufacturer's instructions. ICOs were homogenized in MDA lysis buffer on ice, followed by centrifugation at 13,000 × g for 10 minutes to obtain the supernatant. This supernatant was then incubated with TBA reagent at 95°C for 60 minutes. After cooling to room temperature for 10 minutes, the absorbance was measured at OD 532 nm using a microplate reader. MDA concentrations were determined by calibration with protein quantification (Cheng et al., 2024).

To detect apoptosis in ICOs, they were seeded into a 96-well black-walled, clear-bottom plate and subjected to cold storage. The Caspase-Glo® 3/7 Assay System (Promega, G8090) was followed strictly for the procedure (Jardi et al., 2023). After aspirating the media, 100 μl of thawed CellTiter-Glo® 3D buffer was added to each well. The plate was incubated at room temperature for 1 hour in the dark, with initial shaking for 10 minutes on a microplate shaker. Luminescence was measured using the BioTek SYNERGY NEO2 reader.

**Cell live-dead staining**

ICOs viability was assessed using Calcein AM and PI staining, following the manufacturer’s instructions for the Live-Dead Cell Staining Kit (K2081, Apexbio). ICOs were incubated in a staining solution containing 2 μM Calcein AM and 4 μM PI in HBSS at 37°C for 30 minutes. After incubation, ICOs were washed once with HBSS

to remove unbound dye. Fluorescence microscopy was used to visualize the ICOs, with live cells exhibiting green fluorescence (Calcein AM) and dead cells exhibiting red fluorescence (PI). High-throughput screening was performed using the Opera Phenix High-Content Analysis System (PerkinElmer), and all image analysis was conducted with Harmony software (PerkinElmer) (Pendergraft et al., 2017).

**Mitochondrial ROS determination**

Mitochondrial reactive oxygen species (ROS) production, in correlation with mitochondrial mass, was quantified using the MitoSOX Red mitochondrial superoxide indicator (Yeasen, 40778ES50). Organoids were first washed to remove the complete medium, followed by incubation in a 5 μM MitoSOX working solution for 30 minutes at 37°C in a $CO_2$ incubator. After incubation, the ICOs were gently rinsed with Hank's Balanced Salt Solution (HBSS) and maintained in HBSS for imaging. Red fluorescence, indicative of mitochondrial ROS, was measured using a fluorescence microscope (Kimura et al., 2022). High-content analysis was performed using the Opera Phenix High-Content Analysis System (PerkinElmer), and all image analysis was conducted with Harmony software (PerkinElmer).

**Nuclei preparation for snRNA-seq**

To prepare the nuclei of liver tissues from mice and Syrian hamsters for snRNA-seq, the livers were harvested, washed in cold PBS, blotted dry, and then flash-frozen in liquid nitrogen. The frozen livers are stored at -80°C before use. Homogenize the tissue using the Nuclei PURE Prep Nuclei Isolation Kit (Sigma, NUC-201) following the provided protocol. Filter the homogenate to remove debris and isolate nuclei by centrifugation. Wash the nuclei with a resuspension buffer, filter again to purify, and assess quality using DAPI staining. At last, count the nuclei and proceed with library preparation for sequencing.

For the preparation of ICOs nuclei, ICOs in matrigel were transferred to a 15 ml tube

with 5 ml of cold DMEM/F12 and incubated on ice for 5 minutes to dissolve the matrigel. The mixture was then centrifuged at 500 g for 5 minutes. The supernatant was aspirated, and TrypLE (Gibco, 12604021) was added to dissociate the ICOs at 37°C for 10 minutes. After centrifugation and pellet collection, nuclei were extracted using a dounce homogenizer in NP40 lysis buffer on ice. A 5–10 ml cold NBS solution (1× PBS + 1% BSA + 0.2U/μl RNase Inhibitor) was used to wash the nuclei 3 times. The suspension was then filtered through a 30 μm strainer and centrifuged at 500 g for 5 minutes at 4°C. The supernatant was carefully removed, leaving only the pellet, and the cell count was determined after adding 200 μl of NSB.

**snRNA-seq**

Single-nuclei samples were prepared according to the 10x Genomics Single Cell 3' Reagent Kit v3.1 user guide and established protocols. The single-cell data were subsequently analyzed using 10x Cell Ranger software, version 5.0.1, with reads aligned to the Syrian hamster genome (BCM_Maur_2.0) or the Mouse genome (mm10-2020-A) (Zheng et al., 2017). Seurat package (Seurat_4.0.0) was used to fulfill downstream analyses, including data normalization, variable feature identification, data scaling, linear and non-linear dimensional reduction, and batch-effector correction (Hao et al., 2021). Finally, for snRNA-seq of Syrian hamster and mouse livers, we obtained 26,384 single nuclei, wherein the cut off was set as 200 genes and 200 transcripts detected in each single nucleus. For snRNA-seq of shICOs, 11,928 single nuclei were obtained, and the cut off was set as 500 genes and 1,000 transcripts detected in each single nucleus. Notably, the single-nucleus based data do not involve mitochondrial DNA contaminations.

**Bulk RNA-seq**

RNA from mICOs and shICOs was promptly collected post-hypothermia and rewarming stimulation. The ICOs, embedded in Matrigel, were first washed with cold PBS to eliminate the medium. Lysis was initiated using QIAzol lysing reagent (Qiagen,

Hilden, Germany), and RNA isolation was performed using the RNeasy Mini Kit (Qiagen, 74104) in accordance with the manufacturer's guidelines. Subsequently, libraries were prepared using the VAHTS mRNA-seq V3 Library Prep Kit for Illumina (Vazyme, NR611), adhering to the manufacturer's instructions. The samples were sequenced on the Illumina NovaS4 platform (Mingma, Guangzhou, China), yielding 20-30 million paired-end reads, each 150 nucleotides in length. TrimGalore-0.6.5 was used to trim adapter sequences and low-quality bases from sequencing data. STAR 2.7.5a_2020-06-29 was used to align reads to Syiran hamster genome (BCM_Maur_2.0) and Mouse genome (mm10-2020-A) (Dobin et al., 2013). RSEM-1.3.3 was used to do gene expression quantification (Li and Dewey, 2011). The downstream differential expression was fulfilled by DESeq2_1.28.1 (Love et al., 2014). Pheatmap_1.0.12 was used to generate heatmaps to show the gene expression pattern of interested genes in different experimental groups.

**ICOs fixation and immunocytochemical (ICC) staining**

ICOs were fixed with 4% paraformaldehyde in Dulbecco's Phosphate-Buffered Saline (DPBS) for 45 minutes at room temperature with gentle shaking to prevent adherence. After fixation, the ICOs were washed twice for 15 minutes each with immunofluorescence (IF) buffer, which consists of 0.2% Triton™ X-100, 0.05% TWEEN® 20, and 0.1% BSA in DPBS. Following optional antigen retrieval, cell permeabilization, and blocking, the ICOs were incubated with primary antibodies overnight at 4°C. They were then washed three times for 5 minutes each with IF buffer and incubated with secondary antibodies either overnight at 4°C or for 24 hours at room temperature (Tysoe et al., 2019). After counterstaining with DAPI, the ICOs underwent clearing and mounting for imaging. Images were captured using a 20X objective on a Zeiss LSM 800 confocal laser scanning microscope. The ZEN software was utilized for summing z-stacks and the 3D viewer plugin was employed for generating 3D stereoscopic structures of the ICOs.

**Histology, immunohistochemistry (IHC) and IF techniques**

Mouse, Syrian hamster and rat liver tissues, freshly excised and stored at 4°C, were fixed in 4% paraformaldehyde for adequate preservation. Following fixation, tissues were dehydrated using dimethylbenzene on a HistoCore PEARL processor (Leica), then embedded in paraffin. Paraffin-embedded tissues were sectioned into 4 μm thick slices. For histological analysis, these sections were stained with hematoxylin and eosin (H&E), a standard technique that highlights cellular structures and tissue morphology. Prior to IHC, tissue sections underwent a standardized preparation process, including deparaffinization to remove paraffin, rehydration to restore moisture, and antigen retrieval to expose antigens for subsequent detection.

For IF, livers were fixed in 4% paraformaldehyde for 48 hours and then were dehydrated using a 30% sucrose solution. Subsequently, the tissues were embedded in O.C.T. compound (SAKURA) and flash-frozen in a dry ice-filled container. 10 μm liver sections were cut from the O.C.T.-embedded samples and washed with 0.2% PBST. They were permeabilized in 0.5% Triton X-100 with 10% goat serum for 15 minutes, then blocked with 10% goat serum in PBS for 1 hour. Primary antibodies were applied overnight at 4°C, followed by incubation with secondary antibodies for 2 hours at room temperature. After DAPI counterstaining, the sections were visualized using a Zeiss LSM 800 Confocal Laser Scanning Microscope.

**Antibodies used in this study**

Rabbit anti-KRT19 (1:200, 10712-1-AP, Proteintech), Mouse anti-CD68 (1:50, sc-20060, Santa Cruz), Mouse anti-TNF-α (1:50, sc-133192, Santa Cruz), Rabbit anti-Cleaved Caspase-3 (1:250, 9661, Cell Signaling), Rabbit anti-SOX9 (1:250, 67439-1-Ig, Proteintech), Rabbit anti-SOX9 (1:250, ab185230, Abcam), Mouse anti-KI67 (1:200, 9449, Cell Signaling Technology), Mouse anti-EPCAM (1:200,NBP2-44635, Novus), Rabbit anti- 4HNE (1:200, bs-6313R, bioss), and Rabbit anti-FTH1 (1:250, ab75973, Abcam).

**Statistical analysis**

Statistical examination of data was performed using Prism 10.0 (GraphPad Software).

## References:


BROUTIER, L., ANDERSSON-ROLF, A., HINDLEY, C. J., BOJ, S. F., CLEVERS, H., KOO, B.-K. & HUCH, M. 2016. Culture and establishment of self-renewing human and mouse adult liver and pancreas 3D organoids and their genetic manipulation. *Nature protocols,* 11**,** 1724-1743.

CHENG, W., ZHOU, Y., CHEN, H., WU, Q., LI, Y., WANG, H., FENG, Y. & WANG, Y. 2024. The iron matters: Aged microplastics disrupted the iron homeostasis in the liver organoids. *Science of The Total Environment,* 906**,** 167529.

DOBIN, A., DAVIS, C. A., SCHLESINGER, F., DRENKOW, J., ZALESKI, C., JHA, S., BATUT, P., CHAISSON, M. & GINGERAS, T. R. 2013. STAR: ultrafast universal RNA-seq aligner. *Bioinformatics,* 29**,** 15-21.

GIACALONE, A. G., MERRITT, M. E. & RAGAVAN, M. 2022. Ex Vivo Hepatic Perfusion Through the Portal Vein in Mouse. *Journal of visualized experiments: JoVE***,** 10-3791.

HAO, Y., HAO, S., ANDERSEN-NISSEN, E., MAUCK, W. M., ZHENG, S., BUTLER, A., LEE, M. J., WILK, A. J., DARBY, C. & ZAGER, M. 2021. Integrated analysis of multimodal single-cell data. *Cell,* 184**,** 3573-3587.

JARDI, F., KELLY, C., TEAGUE, C., FOWLER-WILLIAMS, H., SEVIN, D. C., RODRIGUES, D., JO, H., FERREIRA, S., HERPERS, B. & VAN HEERDEN, M. 2023. Mouse organoids as an in vitro tool to study the in vivo intestinal response to cytotoxicants. *Archives of Toxicology,* 97**,** 235-254.

KIMURA, M., IGUCHI, T., IWASAWA, K., DUNN, A., THOMPSON, W. L., YONEYAMA, Y., CHATURVEDI, P., ZORN, A. M., WINTZINGER, M. & QUATTROCELLI, M. 2022. En masse organoid phenotyping informs metabolic-associated genetic susceptibility to NASH. *Cell,* 185**,** 4216-4232.

LI, B. & DEWEY, C. N. 2011. RSEM: accurate transcript quantification from RNA-Seq data with or without a reference genome. *BMC bioinformatics,* 12**,** 1-16.

LOVE, M. I., HUBER, W. & ANDERS, S. 2014. Moderated estimation of fold change and dispersion for RNA-seq data with DESeq2. *Genome biology,* 15**,** 1-21.

PENDERGRAFT, S. S., SADRI-ARDEKANI, H., ATALA, A. & BISHOP, C. E. 2017. Three-dimensional testicular organoid: a novel tool for the study of human spermatogenesis and gonadotoxicity in vitro. *Biology of Reproduction,* 96**,** 720-732.

TYSOE, O. C., JUSTIN, A. W., BREVINI, T., CHEN, S. E., MAHBUBANI, K. T., FRANK, A. K., ZEDIRA, H., MELUM, E., SAEB-PARSY, K. & MARKAKI, A. E. 2019. Isolation and propagation of primary human cholangiocyte organoids for the generation of bioengineered biliary tissue. *Nature protocols,* 14**,** 1884-1925.

ZHENG, G. X. Y., TERRY, J. M., BELGRADER, P., RYVKIN, P., BENT, Z. W., WILSON, R., ZIRALDO, S. B., WHEELER, T. D., MCDERMOTT, G. P. & ZHU, J. 2017. Massively parallel digital transcriptional profiling of single cells. *Nature communications,* 8**,** 14049.

## Supplemental Figure Legends

**Figure S1. The evaluation of tissue injury in mouse and Syrian hamster livers after 4 °C SCS**

(A) H&E staining of mouse and Syrian hamster liver tissues after 1, 3, and 5 days of 4 °C SCS, with freshly resected livers serving as the control group. Fig. 1B shows a partial view of the tissues. Black arrow indicates compromised biliary architectures.

(B) IHC staining for KRT19 in mouse and Syrian hamster liver tissues after 1, 3, and 5 days of 4 °C SCS, with freshly resected livers serving as the control group. Black arrow indicates compromised biliary architectures.

(C) IF staining for KRT19 and CD68 in mouse and Syrian hamster liver tissues after 1, 3, and 5 days of 4 °C SCS, with freshly resected livers serving as the control group. White dash line indicates bile ducts.

**Figure S2. Cross-species comparison between Syrian hamster and mouse cholangiocytes**

(A) Violin plots showing the gene and transcript numbers detected in the snRNA-seq libraries of Syrian hamster and mouse livers.

(B) Cell type proportions of Syrian hamster and mouse livers in the snRNA-seq dataset.

(C) Heatmap showing the DEGs of each cell type in Syrian hamster and mouse livers.

(D) Violin plots showing expression patterns of representative marker genes of each cell type in Syrian hamster and mouse livers.

(E) Heatmap showing the differentially expressed genes (DEGs) between Syrian hamster and mouse cholangiocytes, with representative enriched terms of the

upregulated and downregulated DEGs in Syrian hamster cholangiocytes on the right.
(F) Representative phase-contrast images showing the effect of removing each NFRA composition on the culture of shICOs.

**Figure S3. The evaluation of the culture system of shICOs**

(A) Representative IF microscopy images of mICOs showing the expression of biliary markers SOX9, KRT19 and EPCAM, as well as the proliferation marker KI67.
(B) H&E staining and IHC staining for KRT19 and SOX9 in Syrian hamster liver tissues.
(C) Violin plots showing the gene and transcript numbers detected in snRNA-seq libraries of shICOs.
(D) Heatmap showing the marker genes of hepatocytes and cholangiocytes of both shICOs and Syrian hamster liver tissues in the snRNA-seq dataset.
(E) UMAP projection showing expression patterns of hepatocyte markers (*Hnf4a* and *Tfr2*) and cholangiocyte markers (*Epcam* and *Phhd1*) in the integrated dataset of shICO cells and Syrian hamster liver cells.

**Figure S4. Ferroptosis inhibitor improves the cold preservation of mICOs.**

(A) Scanning of red fluorescence (PI) in shICOs and mICOs under cold stress using a high-content analysis.
(B) Cell viability in mICOs assessed by the ATP assays during the cooling-rewarming process, with treatments of Ferrostatin-1 (50uM) to inhibit ferroptosis or the pan-

caspase inhibitor Z-VAD(OH)-FMK (50uM) to inhibit apoptosis. Data are presented as mean ± standard deviation. ****P < 0.0001; ***p < 0.001; *P < 0.05; n.s. p > 0.05, not significant (t-test).

**Figure S5. shICOs exhibit more efficient iron homeostasis for anti-ferroptosis**

(A) Diagram illustrating bulk RNA-seq of mICOs and shICOs at 6 time points during the cooling-rewarming process (n = 2 biological replicates).

(B) Heatmap showing the expression patterns of key ferroptosis-related marker genes for both mICOs and shICOs across 6 time points during the cooling-rewarming process.

(C) H&E staining and IF staining for 4HNE, TNF-α and CD68 showing improved cold preservation of mouse bile ducts in UW solution with deferoxamine (DFO, 1mM) after 5 days of 4 °C SCS.

**Figure S6. H&E staining and IF staining for CC3, 4HNE, CD68 and TNF-α showing improved cold preservation of rat bile ducts in UW solution with deferoxamine (DFO, 100 μM and 1mM) after 5 days of 4 °C SCS. Black arrow indicates compromised biliary architectures, whereas star indicates undamaged biliary architectures.**

**Supplemental Table Legends**

**Table S1. Marker genes of each liver cell type in Syrian hamster and mouse snRNA-seq data**

**Table S2. DEGs between Syrian hamster and mouse cholangiocytes in snRNAs-seq data**

**Table S3. Maker genes of cholangiocytes and hepatocytes in both shICO and Syrian hamster snRNA-seq data**

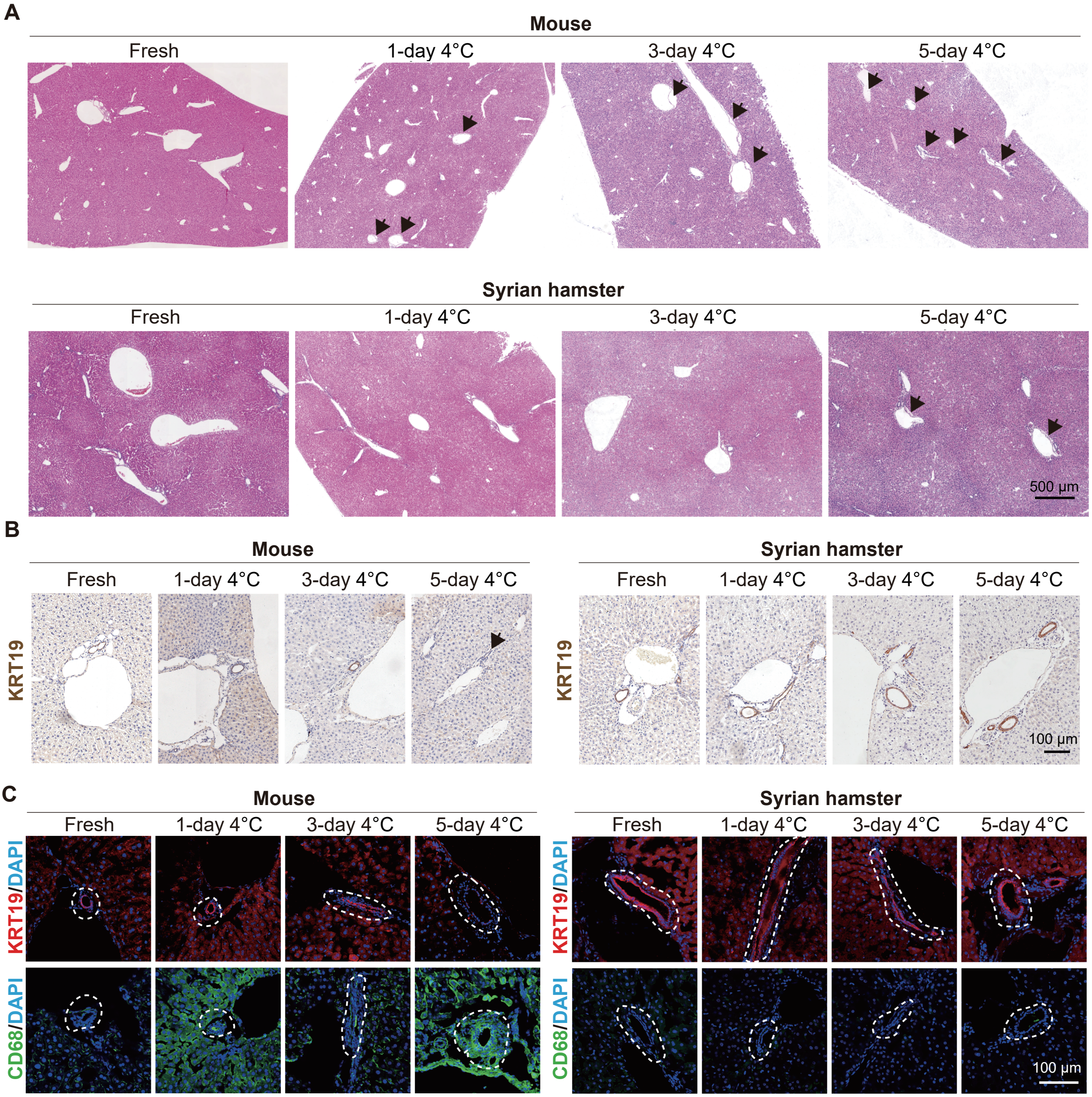
A
Mouse
Fresh
1-day 4°C
3-day 4°C
5-day 4°C
Syrian hamster
Fresh
1-day 4°C
3-day 4°C
5-day 4°C
500 μm
B
Mouse
Fresh
1-day 4°C
3-day 4°C
5-day 4°C
KRT19
Syrian hamster
Fresh
1-day 4°C
3-day 4°C
5-day 4°C
KRT19
100 μm
C
Mouse
Fresh
1-day 4°C
3-day 4°C
5-day 4°C
KRT19/DAPI
CD68/DAPI
Syrian hamster
Fresh
1-day 4°C
3-day 4°C
5-day 4°C
KRT19/DAPI
CD68/DAPI
100 μm

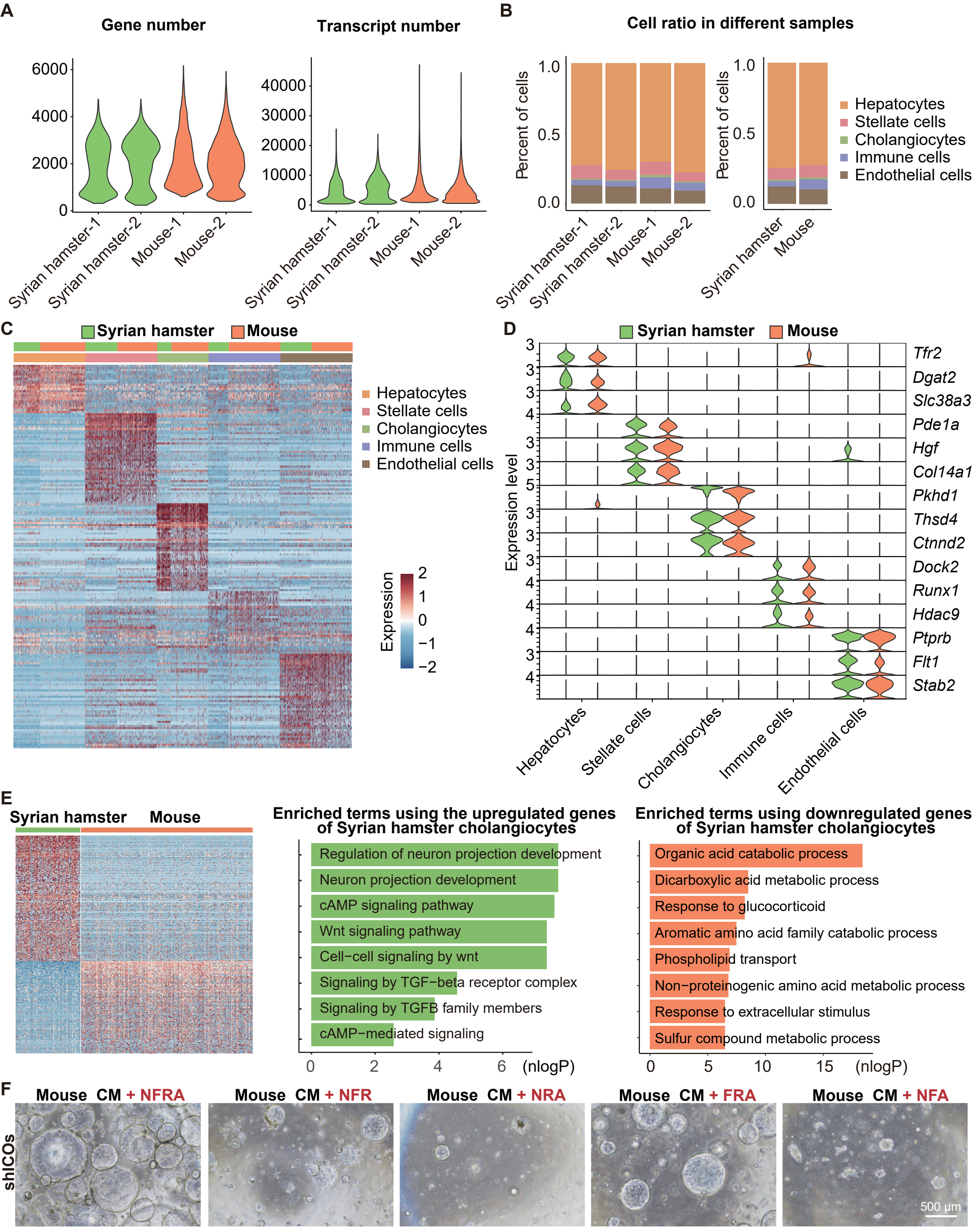

A
Gene number
Transcript number
Syrian hamster-1
Syrian hamster-2
Mouse-1
Mouse-2
B
Cell ratio in different samples
Percent of cells
Syrian hamster
Mouse
Hepatocytes
Stellate cells
Cholangiocytes
Immune cells
Endothelial cells
C
Syrian hamster
Mouse
Expression
D
Expression level
Tfr2
Dgat2
Slc38a3
Pde1a
Hgf
Col14a1
Pkhd1
Thsd4
Ctnnd2
Dock2
Runx1
Hdac9
Ptprb
Flt1
Stab2
E
Enriched terms using the upregulated genes of Syrian hamster cholangiocytes
Regulation of neuron projection development
Neuron projection development
cAMP signaling pathway
Wnt signaling pathway
Cell−cell signaling by wnt
Signaling by TGF−beta receptor complex
Signaling by TGFB family members
cAMP−mediated signaling
(nlogP)
Enriched terms using downregulated genes of Syrian hamster cholangiocytes
Organic acid catabolic process
Dicarboxylic acid metabolic process
Response to glucocorticoid
Aromatic amino acid family catabolic process
Phospholipid transport
Non−proteinogenic amino acid metabolic process
Response to extracellular stimulus
Sulfur compound metabolic process
(nlogP)
F
Mouse CM + NFRA
Mouse CM + NFR
Mouse CM + NRA
Mouse CM + FRA
Mouse CM + NFA
shICOs
500 μm

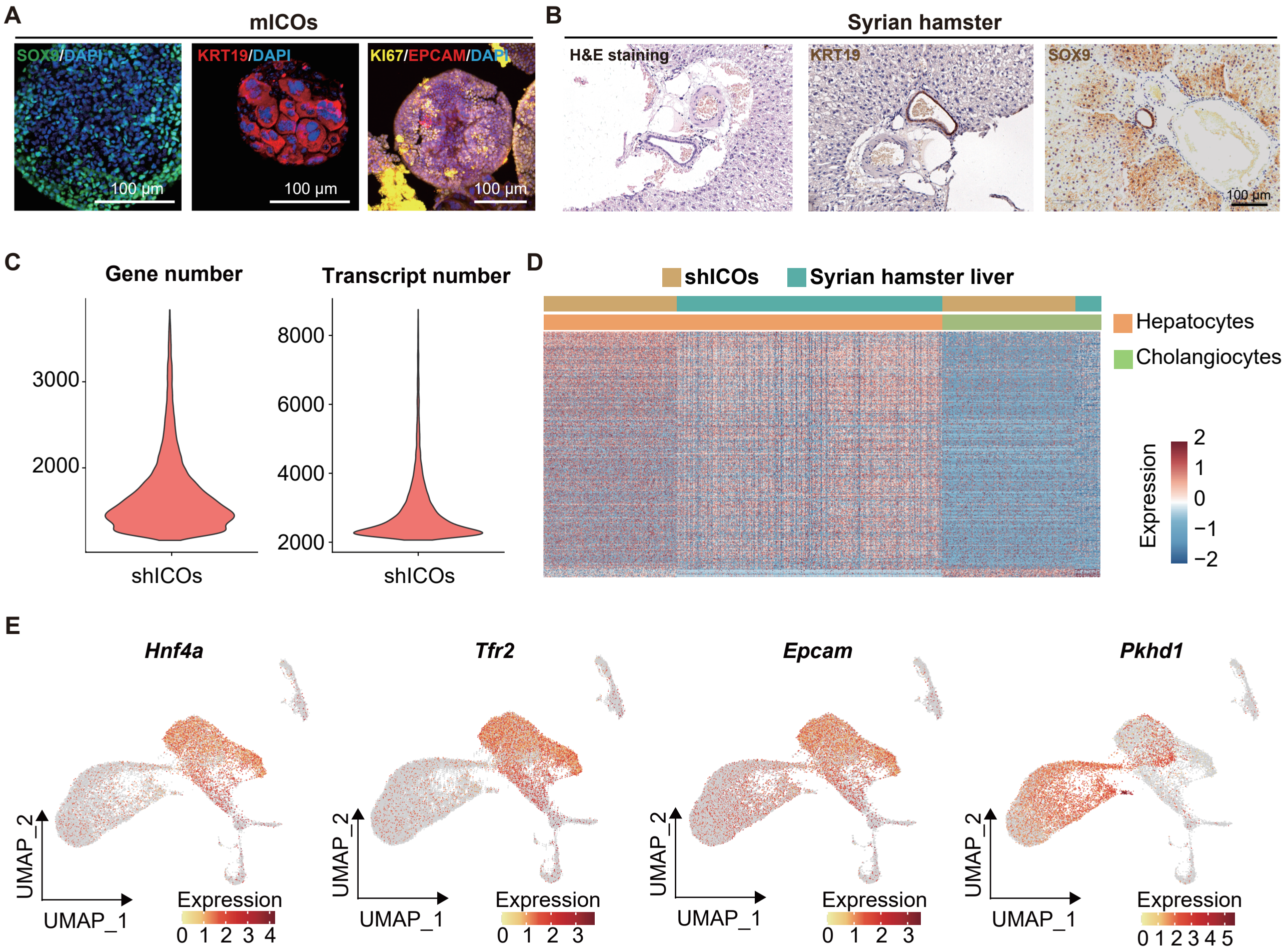

A
mICOs
SOX9/DAPI
KRT19/DAPI
KI67/EPCAM/DAPI
100 μm
100 μm
100 μm
B
Syrian hamster
H&E staining
KRT19
SOX9
100 μm
C
Gene number
Transcript number
3000
2000
8000
6000
4000
2000
shICOs
shICOs
D
shICOs
Syrian hamster liver
Hepatocytes
Cholangiocytes
Expression
2
1
0
−1
−2
E
Hnf4a
Tfr2
Epcam
Pkhd1
UMAP_2
UMAP_1
Expression
0 1 2 3 4
0 1 2 3
0 1 2 3
0 1 2 3 4 5

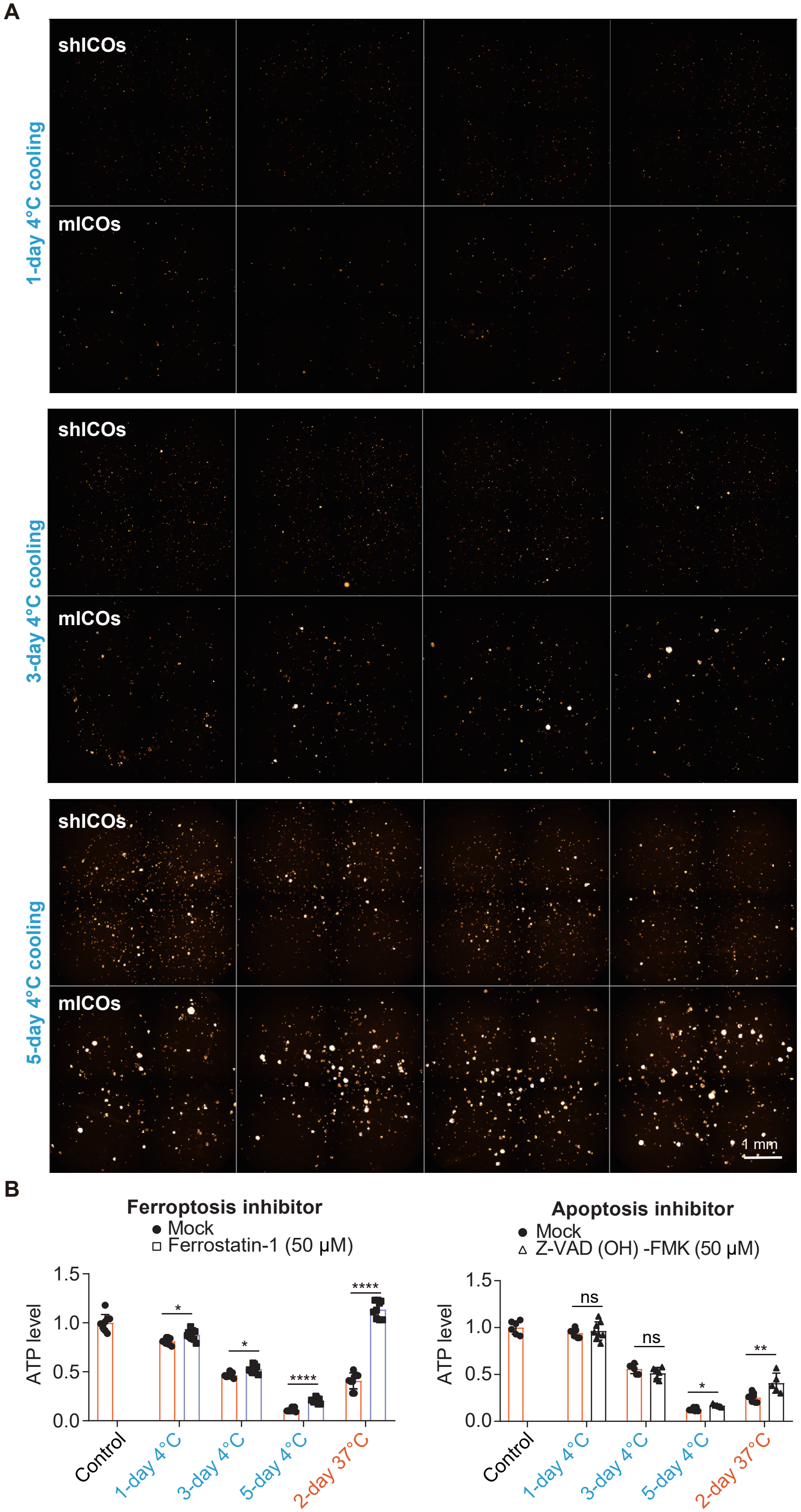
A
1-day 4°C cooling
shICOs
mICOs
3-day 4°C cooling
shICOs
mICOs
5-day 4°C cooling
shICOs
mICOs
1 mm
B
Ferroptosis inhibitor
Mock
Ferrostatin-1 (50 μM)
ATP level
1.5
1.0
0.5
0.0
*
*
****
****
Control
1-day 4°C
3-day 4°C
5-day 4°C
2-day 37°C
Apoptosis inhibitor
Mock
Z-VAD (OH) -FMK (50 μM)
ATP level
1.5
1.0
0.5
0.0
ns
ns
*
**
Control
1-day 4°C
3-day 4°C
5-day 4°C
2-day 37°C

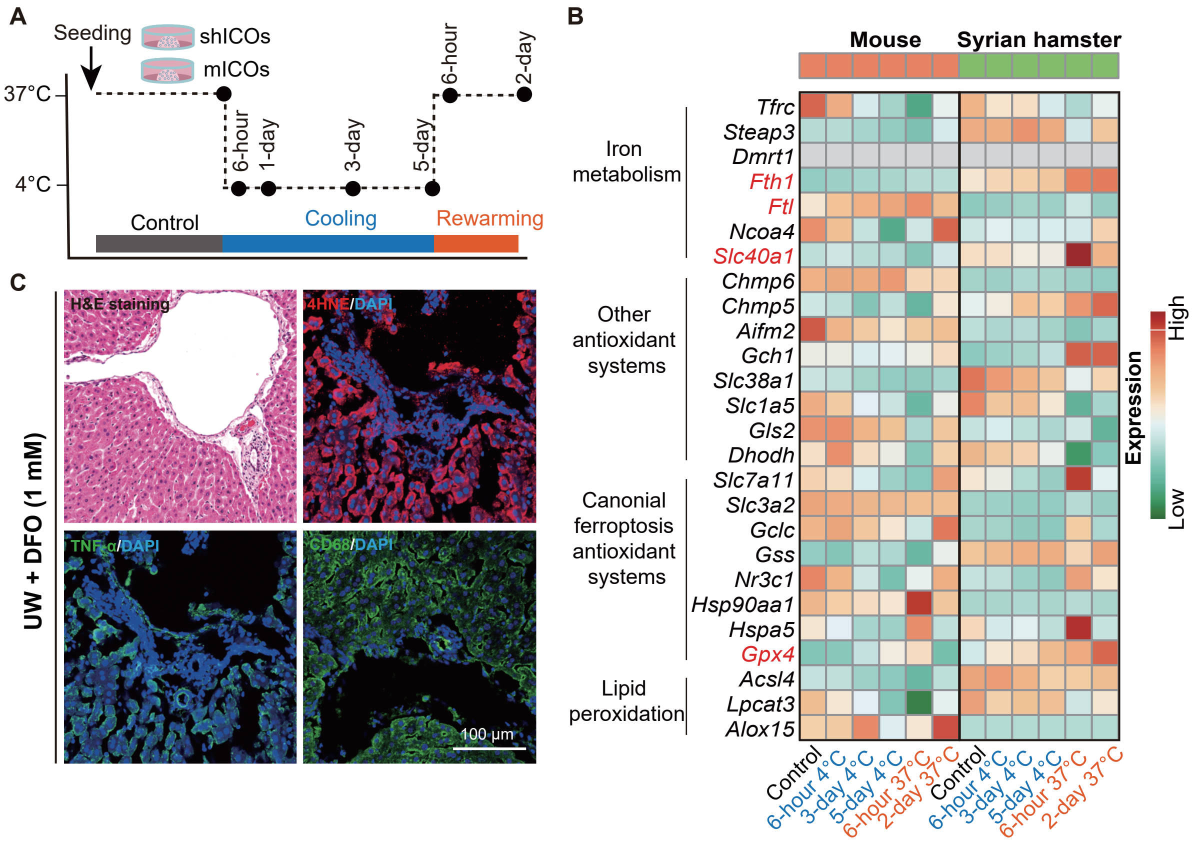

A
Seeding
shICOs
mICOs
37°C
4°C
6-hour
1-day
3-day
5-day
6-hour
2-day
Control
Cooling
Rewarming
B
Mouse
Syrian hamster
Iron metabolism
Other antioxidant systems
Canonial ferroptosis antioxidant systems
Lipid peroxidation
Tfrc
Steap3
Dmrt1
Fth1
Ftl
Ncoa4
Slc40a1
Chmp6
Chmp5
Aifm2
Gch1
Slc38a1
Slc1a5
Gls2
Dhodh
Slc7a11
Slc3a2
Gclc
Gss
Nr3c1
Hsp90aa1
Hspa5
Gpx4
Acsl4
Lpcat3
Alox15
Control
6-hour 4°C
3-day 4°C
5-day 4°C
6-hour 37°C
2-day 37°C
Control
6-hour 4°C
3-day 4°C
5-day 4°C
6-hour 37°C
2-day 37°C
Expression
High
Low
C
UW + DFO (1 mM)
H&E staining
4HNE/DAPI
TNF-α/DAPI
CD68/DAPI
100 μm

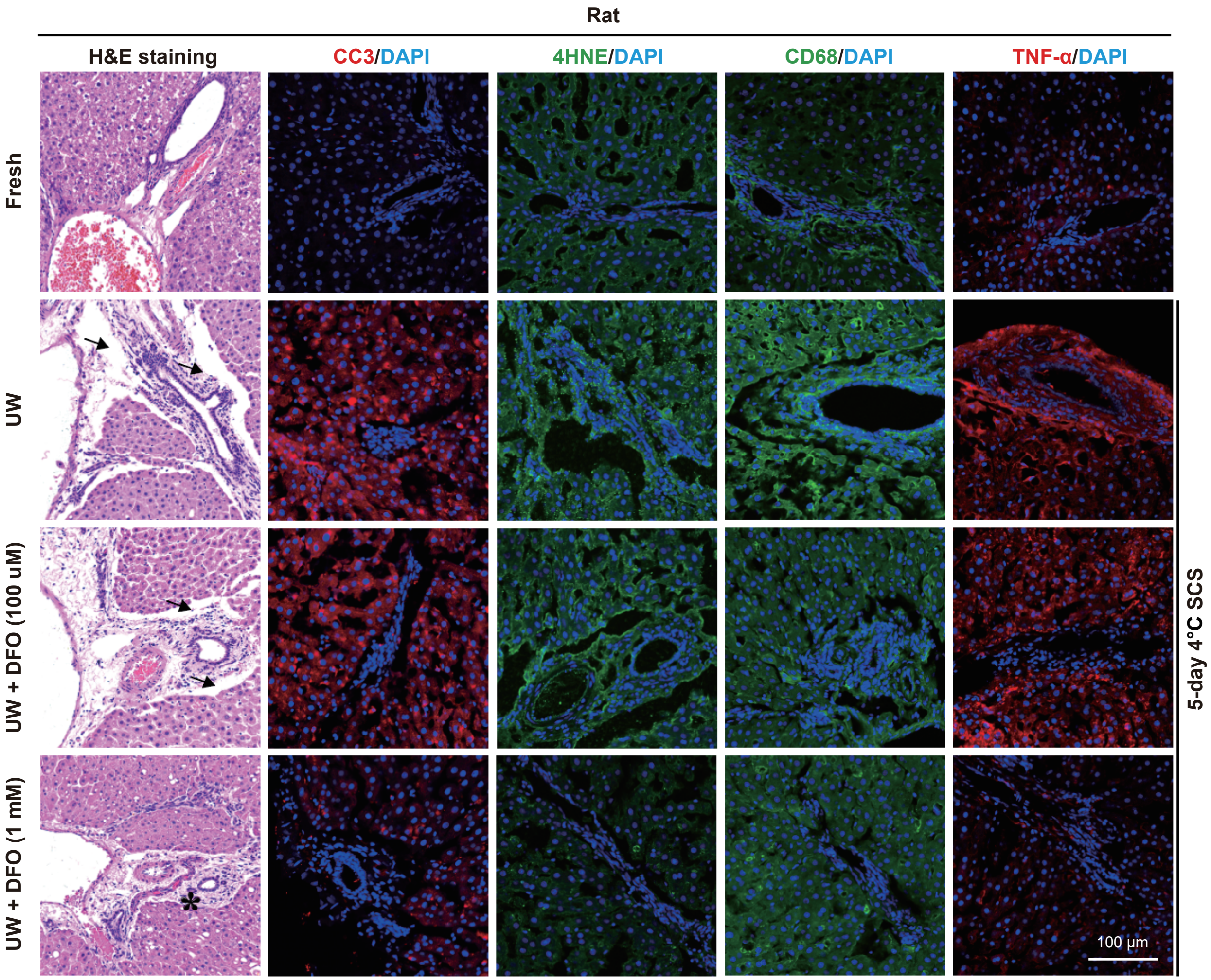

Rat
H&E staining
CC3/DAPI
4HNE/DAPI
CD68/DAPI
TNF-α/DAPI
Fresh
UW
UW + DFO (100 uM)
UW + DFO (1 mM)
5-day 4°C SCS
100 μm
